# Optimizing Exposure Measures in Large-Scale Household Air Pollution Studies: Results from the Multicountry HAPIN Trial

**DOI:** 10.1021/acs.est.4c08052

**Published:** 2025-01-14

**Authors:** K Steenland, A Pillarisetti, M Johnson, J Rosenthal, K Balakrishnan, L Underhill, L Thompson, J McCracken, L Waller, L Nicolaou, M Clark, W Checkley, J Peel, T Clasen

**Affiliations:** † Rollins School of Public Health, Emory U, Atlanta, Ga 30322, United States; ‡ University of California at Berkeley, Berkeley, California 94720, United States; § Berkeley Air, Berkeley, California 94704, United States; ∥ Fogarty International Center, National Institute of Health, Bethesda, DC 20892, United States; ⊥ Faculty of Public Health, SRI Ramachandra Medical College, Chennai 60056, India; # School of Medicine, Washington U, St. Louis, Missouri 63110, United States; ∇ School of Nursing, Emory U, Atlanta, Ga 30322, United States; ○ College of Public Health, University of Georgia, Athens, Ga 30602, United States; ◆ Environmental Health and Engineering, Johns Hopkins U, Baltimore, Maryland 21205, United States; ¶ Colorado School of Public Health, Colorado State University, Ft. Collins, Colorado 80045, United States; k School of Medicine, Environmental Health, Johns Hopkins Sch. of Public Health, Baltimore, Maryland 21205, United States

**Keywords:** household air pollution, personal samples, repeated measures, PM_2.5_

## Abstract

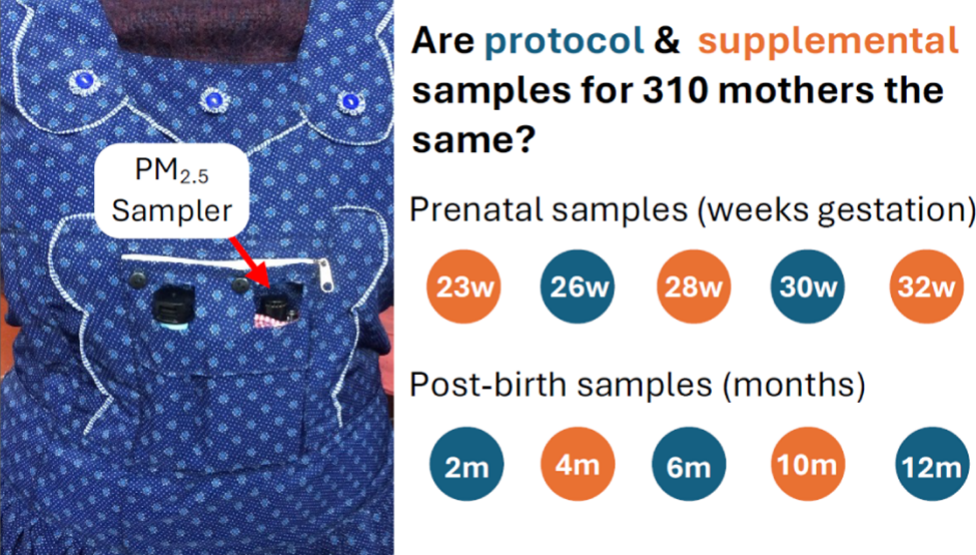

Repeated measurements of household air pollution may provide better estimates of average exposure but can add to costs and participant burden. In a randomized trial of gas versus biomass cookstoves in four countries, we took supplemental personal 24-h measurements on a 10% subsample for mothers and infants, interspersed between protocol samples. Mothers had up to five postrandomization protocol measurements over 16 months, while infants had three measurements over one year. For the subsample, we added up to 6 supplemental postrandomization samples for mothers and 3 for infants, measuring PM_2.5_, black carbon (BC) (mothers only), and carbon monoxide (CO) at each visit. 310 mothers had both protocol (*n* = 1026) and supplemental (*n* = 1099) valid exposure measurements. For children, supplemental data sufficient for analysis were collected in only two countries; 94 infants had both protocol (*n* = 317) and supplemental (*n* = 234) samples. The geometric means for protocol and supplemental samples for mothers for PM_2.5_ were 37 μg/m^3^ and 38 μg/m^3^, respectively, while for infants, they were 42 μg/m^3^ and 46 μg/m^3^. Mixed models comparing supplemental to protocol samples, controlling for covariates, found few differences between protocol and supplemental samples. Supplemental analyses among control mothers with complete protocol measurements found that an average of three measurements explained 81% of the variance of the average of all six measurements.

## Abstract 2

This paper describes the type of sampling strategy in a study of household air pollution needed to accurately estimate long-term average exposure.

## Introduction

Globally, nearly 3 billion people burn solid fuels or biomass (e.g., wood, dung, charcoal) in inefficient and poorly vented combustion devices (i.e., open fires, traditional stoves) to meet daily cooking needs.^1^ The resulting household air pollution (HAP) is a leading risk factor for global morbidity and mortality.^2^ There are relatively few studies with quantitative data on personal exposures to HAP; many studies have relied on imprecise, proxy exposure measures.^3^ Direct measures of fine particulate matter (PM_2.5_) exposure have been particularly challenging due to the limitations of affordable, appropriate, feasible, and reliable instrumentation.^4−6^ Furthermore, while repeated measurements may better characterize long-term exposure, they are costly and labor-intensive and impose a higher burden on participants. A gap exists in the literature regarding the optimal number of measurements needed to accurately characterize long-term average exposures in resource-limited settings, where solid fuel use is common.

The Household Air Pollution Intervention Network (HAPIN) trial was a four-country (Guatemala, India, Peru, Rwanda) randomized controlled trial (RCT) evaluating the effects of a free liquefied petroleum gas (LPG) cookstove and fuel intervention versus cooking on traditional biomass stoves among 800 households (split equally between control and intervention arms) in each of the four countries, for a total of 3200 households.^7^

In the HAPIN trial, we measured personal 24-h exposure to PM_2.5_, black carbon, and carbon monoxide (CO) at multiple time points for three study populations of interest: pregnant mothers, their infants, and older adult women (aged 40–79 years). All three groups came from the same households. Following protocol, we collected up to six measurements of pregnant mothers (one at baseline prior to randomization and stove installation, two at follow-up during pregnancy, and three following birth); three among infants in their first year of life; and up to six measurements in older adult women during the approximately 18 months of observation. Here, we consider only the samples from mothers and infants (see Table 1), as the sample of older adult women was much smaller (they were recruited in about 15% of households).

**Table 1:** Schedule of 24-h Personal Sampling for Mothers and Their Infants in the HAPIN Trial

	pregnant woman/mother	pregnant woman/mother	infant	infant
gestation or child age	protocol sample	supplemental sample	protocol sample	supplemental sample
<20 weeks (baseline, prerandomization)	X			
23 weeks		X		
26 weeks	X			
28 weeks		X		
30 weeks	X			
32 weeks		X		
2 months old	X		X	
4 months old		X		X
6 months old	X		X	
8 months old		X		X
10 months old		X		X
12 months old	X		X	

The LPG stove and fuel intervention was successful in lowering exposures. Exposure reductions were consistent over time and were similar across research locations.^8^Figure S1 shows the sampling results for PM_2.5_ for mothers. Similar exposure reductions were seen for children.^9^

We sought to determine whether our per-protocol exposure estimates accurately classify long-term exposure averages, given the potentially high day-to-day and seasonal variability in exposures within homes where biomass use is common. To do this, we doubled the number of assessments in a random subsample of approximately 10% of the study participants per study site. The random sample was selected monthly among newly recruited households. The purpose of these supplemental measurements was to determine whether their averages were comparable to the averages of the smaller number of per-protocol measurements for the same individuals. If so, it would suggest that the average of the per-protocol measurements would be a reasonable estimate of the long-term exposure for the study participants.

While it is recognized that it is best to have multiple measurements of exposure in follow-up studies, there is limited information on the number of measurements necessary to secure adequate estimates of long-term exposure. Here, we describe the results of the HAPIN supplemental sampling compared to the protocol sampling.

## Methods

Details on the study settings and population have been previously described.^8^ The trial was undertaken in low-density rural communities where households relied primarily on biomass (wood, charcoal, dung, and agricultural residue) for cooking and where other sources of air pollution exposure were minimal. The trial sites varied in altitude and climate, spanning from high-altitude regions in Puno, Peru, to low-altitude regions in Tamil Nadu, India, and intermediate-altitude regions in Jalapa, Guatemala, and Kayonza, Rwanda. Women were recruited early in the pregnancy from local clinics. To be eligible, pregnant women were required to be 19–34 years old, 9-19 weeks pregnant (confirmed by ultrasound), and nonsmokers.

Households were randomized between those receiving gas stoves and free fuel and control households, which continued using biomass stoves. Those receiving gas stoves were followed to determine if they were using the intervention and not biomass stoves. Adherence was high, with minimal evidence of stove stacking (the use of both gas and biomass stoves at the same time).^10^ Control households were given gas stoves or a package of equivalent value at the end of the study. Compliance with wearing PM_2.5_ monitors was also reasonable; instruments were worn 61% of daytime hours.^8^ Samples were not excluded for wearing compliance because participants were asked to keep instruments near them when unable to wear the devices.

Planned protocol sampling was conducted at 6 time points for pregnant mothers (at baseline before randomization and stove installation plus two additional time points pre- and postpregnancy) and 3 times for infants (Table 1).

Full description of exposure sampling methods can be found in Johnson et al.^11^ Briefly, for adults, we used the Enhanced Children’s MicroPEM (ECM, RTI International, USA), a robust, lightweight, and validated combined gravimetric and nephelometric PM_2.5_ monitor, and the passive Lascar CO logger (Lascar Electronics, UK). The ECM was equipped with a 2.5 μm size-cut impactor and a flow rate of 0.3 L per minute. PM_2.5_ sampling was done using Teflon filters, which were weighed pre- and postsampling. BC was estimated on PM_2.5_ filters using an OT21 transmissometer (Magee Scientific, USA). CO measurements were logged at 1-min intervals; the Lascar logger has a range of 0–300 ppm. All measurements were for 24 h.

For infants aged <1 year, we used a validated indirect assessment method, based on measuring area samples in the environments where infants spent time, and assessing the amount of time spent in each of these areas.^12^ Indirect assessment was needed due to the difficulty in equipping infants with ECM monitors. This method takes advantage of a combination of small, coin-sized proximity sensors (Proximity, USA) worn by study infants along with microenvironmental pollutant measurements made with the ECM in the most commonly occupied rooms, outside near the home, and on their mother. The child’s location was determined by detecting the Bluetooth signal from the proximity sensors with receivers collocated alongside microenvironmental monitors. Personal exposures for the child were estimated by integrating the corresponding measured area concentrations with time spent in the respective locations.

For a random 10% of study participants, we took supplemental samples for mothers and their infants, which were collected using the same methods described above, at approximate midpoints between the protocol samples (see Table 1). This resulted in a total of up to 6 supplemental samples for mothers across follow-up and up to 3 supplemental samples for their infants. The HAPIN study implemented rolling (ongoing) recruitment over 15 months; hence, both protocol and supplemental measurements were randomly dispersed across seasons. We chose a 10% sample (320 mothers and 320 children) prior to study initiation as an estimate of what a reasonable number of extra samples would be sufficient to evaluate whether they were comparable to the protocol samples, with reasonable extra costs and associated fieldwork. We applied for and used supplemental funds to finance these supplemental measurements.

Descriptive statistical analysis consisted of comparing mean pollutant levels for protocol samples with the means of supplemental samples for participants who had both measures. In addition, we used mixed models to regress pollutants on an indicator variable for protocol versus supplemental samples with a random effect for participants. A random effect for households accounts for the correlation (nonindependence) of repeated measures taken across the same period. An indicator variable for protocol versus supplemental samples indicated the difference in pollutant exposure levels between the two types of samples (on the log scale), which was our main result of interest. Covariates were chosen a priori based on the literature^4,14^ and our own experience.^8,9^ We chose the most important time-independent predictors of exposure—study arm and study location—that were the same across women within those strata but conceivably could have differed somewhat between protocol and supplemental samples. We chose time-dependent variables for which we had reliable data: season (winter or not), weekend, and pre- vs postgestation (for mothers). Time-dependent variables were of interest in that the supplemental samples could differ in these predictors compared with protocol samples, although the rolling recruitment made this unlikely. Winter months in Guatemala and India were November–February, while in Peru they were May–August. Rwanda showed little temperature variation and hence was coded as without winter months.

Analyses of mothers’ samples were restricted to the postrandomization period to ensure comparability, as mothers’ samples in the intervention arm were markedly higher at the prerandomization (baseline) measurement, when biomass was in use, than after gas stove installation. Pollutants were log-transformed for regression models to better approximate normality (a constant of 0.01 was added to the CO values that were 0).

We generated QQ plots using deciles of the distributions to compare protocol and supplemental samples for mothers and infants using deciles up to the 90th, as the maximum at 100% was subject to the influence of outliers. QQ plots provide a visual comparison of the protocol and supplemental samples by deciles to enable a comparison across the entire distribution, rather than just a summary mean or median (if the deciles are completely concordant, then both lines of the graph will be superimposed, with a 45-degree slope, while deviations of the two lines indicate discordance). Discordant QQ plots might indicate that the distributions of protocol and supplemental samples could differ even if their means were similar.

Analyses of supplemental data for children were restricted to Guatemala and India, where 94 kids (54 in Guatemala, 40 in India) had valid supplemental samples for PM_2.5_, while in Peru, no children had supplemental samples, and in Rwanda, only 10 did. The lack of supplemental samples from Peru and Rwanda resulted from unexpected problems in data transmission and processing.

As a sensitivity analysis, we used the mothers’ PM_2.5_ value to replace the child’s PM_2.5_ estimate when the child’s value was missing, given the high correlation between mother and child analyses (Spearman’s rho ∼0.89), which was expected given that the infants spent most of their time with their mothers. This greatly increased the sample size and included Peru and Rwanda in the analysis.

We also conducted additional supplementary analyses of samples from 251 control mothers who had all six protocol samples to calculate the *R*^2^ for predicting the overall individual means across the six samples, using either randomly chosen 1, 2, 3, or 4 samples of their six samples. In these analyses only, there was no adjustment for covariates.

## Results

For mothers in all 4 countries and children in Guatemala and India, we fulfilled the goal of including an additional 10% in the supplemental sampling. However, many participants with both protocol and supplemental samples had missing (no sample taken) or invalid (failure of equipment or lab errors) measurements. Exposure data were missing/invalid for PM_2.5_, BC, and CO for 27%, 32%, and 29% of the total number of mothers’ samples, respectively; the percent missing was similar for both protocol and supplemental visits. For infants, 49% of PM_2.5_ data and 52% of CO data were missing, with higher numbers partly because the follow-up period for infants often corresponded to the pandemic, when field visits were more difficult. Again, the percent missing for infants was similar between the protocol and supplemental samples. In the text below, we describe “valid measurements” as those which were neither missing nor invalid.

Results for the mothers and their infants who had both protocol and supplemental measurements (310 mothers and 153 infants) are shown in Tables 2 and 3. For mothers, both the arithmetic and geometric means of the three pollutants were quite similar between the protocol and supplemental samples. For infants, the arithmetic means differed somewhat, although not markedly, but geometric means were very similar. This suggests that outliers in the untransformed distribution are largely responsible for the difference as the geometric means reduce the influence of outliers. Further comparisons of the distributions can be seen in QQ plots (FiguresS2–S8), which show that for mothers, the protocol versus supplemental samples for PM2.5 were quite similar, so the plot approximates a straight line at a 45-degree angle. For infants, the same is true until the upper deciles, where the supplemental samples have somewhat higher values than the protocol samples. A QQ plot in Figure S4 for log-transformed infants’ PM_2.5_ shows better agreement for infants, as might be expected for environmental measurement, which are usually log-normally distributed (and which correspond to our mixed models, where we model log-transformed pollutants). QQ plots for mothers for BC and CO, and for infants for CO and log CO, are shown in Figures S5–S8. The general concordance of the decile cut-points implies that the overall distributions of protocol and supplemental samples were similar.

**Table 2:** Postrandomization Samples for 310 Mothers Who Had Both Protocol and Supplemental Samples^a^

Pollutant	Type	Mean	SD	GM	GSD	Min	Max	*N*
PM_2.5_ (μg/m^3^)	Protocol	66.2	103	36.6	2.7	4	1059	1026
	Supplemental	65.2	94.1	37.6	2.7	4	1041	1099
BC (μg/m^3^)	Protocol	6.6	7.8	4.3	2.5	0.7	93.9	878
	Supplemental	6.9	7.8	4.4	2.5	0.9	81.5	1088
CO (ppm)	Protocol	1.7	4.6	0.31	8.2	0	71.8	1017
	Supplemental	1.5	3.4	0.29	8.6	0	55	1063

a 310 out of 3195 mothers were chosen for the supplemental sampling. 295, 292, and 285 had at least one valid PM_2.5_, BC, or CO protocol and supplemental measurements, respectively. Baseline prerandomization samples were excluded, ensuring comparability within arm across measurements. A constant of 0.01 was added to CO samples to avoid taking any logs of 0. SD = standard deviation; GM = geometric mean; GSD = geometric standard deviation.

**Table 3:** Samples for 94 Infants Age 0–1 Year Who Had Both Protocol and Supplemental Samples*

Pollutant	Type	Mean	SD	GM	GSD	Min	Max	*N*
PM_2.5_ (μg/m^3^)	Protocol	64.1	72.8	41.7	2.4	10	130	190
	Supplemental	79.3	111.0	46.7	2.7	10	752	183
CO (ppm)	Protocol	1.18	2.5	0.23	8.3	0	25	190
	Supplemental	1.27	3.4	0.23	7.6	0	35	161

* 153 infants in Guatemala and India were in the randomly chosen subsample, of which 94 had both valid protocol and supplemental measurements for PM and 83 had valid protocol and supplemental samples for CO. Rwanda and Peru were not included here as they had few or no valid supplemental samples. Black carbon measurements were not available for infants. Constants of 0.01 were added to CO prior to taking logs. SD = standard deviation; GM = geometric mean; GSD = geometric standard deviation.

We also calculated the average difference between the mean of protocol measurements and the mean of supplemental measurements for individual women who had at least one of each. For PM_2.5_, among the 295 women who had at least one measurement of each, the mean difference was 3.7 μg/m^3^, which was not significantly different from 0 (*p* = 0.38). For BC, the mean difference was −0.11 μg/m^3^ across 292 women, not significantly different from 0 (*p*= 0.74). For CO, the mean difference was 0.07 ppm across 285 women, also not significantly different from 0 (*p* = 0.68). For children, a similar calculation found a higher average difference between the mean of regular and extra samples of PM_2.5_ of 12.4 μg/m^3^, which nonetheless was not statistically significant at the 0.05 level (*p* = 0.30). For children, for CO, the difference was only 0.03 (*p* = 0.91).

We compared protocol to supplemental measurements using mixed models while adjusting for study site, study arm, and season (winter or not), weekend, and pre vs postbirth for mothers and including a random intercept to account for the nonindependence of estimates from the same subjects. Tables 4 and 5 show the key results, with the coefficient for supplemental versus protocol samples indicating the average difference between them. We also show the effect of time-varying variables, which, if uncontrolled, might distort differences between supplemental and protocol samples.

**Table 4:** Model Results for Mothers for Log-Transformed Pollutants^a^

Pollutant^b^	Coefficient	Standard Error	*t* Value	*p-*Value	ICC
PM**_2.5_** (*n* = 2125)^c^^c^					
Supplemental	0.016	0.032	0.51	0.61	0.22
Study arm^d^	0.875	0.054	16.06	<.0001	
Winter	0.11̀	0.039	2.84	0.005	
Weekend	–0.192	0.083	–2.29	0.02	
Prebirth vs postbirth	0.162	0.033	4.97	<.0001	
**BC** (*n* = 1966)^c^^c^					
Supplemental	0.011	0.027	0.41	0.68	0.27
Study arm^d^	0.898	0.049	18.27	<.0001	
Winter	0.138	0.034	3.99	<.0001	
Weekend	–0.132	0.0691	–1.9	0.06	
Prebirth vs postbirth	0.107	0.028	3.81	0.0001	
**CO** (*n* = 2080)^c^^c^					
Supplemental	–0.173	0.0800	–2.17	0.03	0.14
Study arm^d^	1.514	0.116	13.03	<.0001	
Winter	–0.125	0.098	–1.28	0.20	
Weekend	0.0123	0.188	0.07	0.95	
Prebirth vs postbirth	0.362	0.080	4.52	<.0001	

a 310 mothers had both protocol and supplemental measurement. Log-transformed pollutants were regressed on an indicator variable for supplemental vs protocol samples and on study arm, study site, whether the sample was taken in winter or not (for Guatemala, Peru, India; winter and nonwinter temperature does not differ in temperature in Rwanda), and weekend or not.

b All pollutant variables log transformed.

c Number of valid measurements for the mothers.

d Intervention arm is the referent.

**Table 5:** Model Results for 94 Infants, for Log-Transformed Pollutants, from India and Guatemala^a^

Pollutant^b^	Coefficient	Standard Error	*t* Value	*p-*Value	ICC
**PM2.5** (*n* = 373)^c^^c^					0.40
supplemental	0.061	0.067	0.92	0.36	
Study arm^d^	1.045	0.104	9.98	<.0001	
Winter	0.062	0.070	0.88	0.40	
**CO** (*n* = 351)^c^^c^					0.25
Supplemental	0.027	0.175	0.15	0.88	
Study arm^d^	2.197	0.231	9.52	<.0001	
Winter	0.0617	0.0700	0.88	0.38	

a Excludes Peru and Rwanda, where infants had no or too few valid supplemental measurements. Log-transformed pollutants were regressed on an indicator variable for supplemental vs protocol samples and on study arm, study site, and winter vs no winter. There were too few samples on weekends to add to this model.

b All pollutant variables log transformed.

c Number of valid measurements for the children who had both protocol and supplemental measurements.

d Intervention arm is the referent.

There were no significant differences between protocol and supplemental samples at the *p* ≤ 0.05 level, except for CO for mothers, where the difference of protocol vs supplemental samples for log CO had a *p*-value of 0.03 (though this increased to 0.06 after removing the top 1% of CO observations). We note that there was only an 8% difference on the log scale for the CO protocol and supplemental samples. As expected, for both mothers and children, there were strong differences between control and intervention arms in the expected direction whereby control samples were predicted to be higher by the model. There were also notable differences between the study sites (data not shown). For mothers, winter months had higher PM_2.5_ and black carbon levels but lower CO levels, and PM_2.5_ and BC were lower on weekends. These trends for season and weekends were less apparent in the children’s samples; we saw similar findings in sensitivity analysis using replacement for missing children’s data. For mothers, prebirth levels were significantly higher than postbirth levels, possibly reflecting spending less time in the kitchen after birth.

In the sensitivity analysis, in which we replaced missing child’s data for PM_2.5_ with the mothers’ PM_2.5_ levels, we found again that the supplemental samples did not differ from the protocol samples (*p* = 0.31, Table S1). In this analysis, the number of samples analyzed (for children with both protocol and supplemental samples) increased greatly from 373 to 1211 for 241 children (vs 153 children when not using imputation), and we were able to include Peru and Rwanda. The amount of nonmissing data in the original sample was 51% in Guatemala and India, the two included countries. After imputation, we had 82% nonmissing data, including all four countries.

In the mixed-model regression using log-transformed exposures, intraclass correlation coefficients for models of mothers’ data were 0.22, 0.27, and 0.14 for PM_2.5_, BC, and CO_2_, respectively (Table 4). These differed little for protocol vs supplemental samples; e.g., for PM_2.5_, the ICC was 0.23 for protocol samples and 0.22 for supplemental samples. For infants, the ICCs were 0.40 and 0.25 for PM_2.5_ and CO_2_, respectively (Table 5). These low ICCs indicate that variation within samples (across samples in the same households) was greater than variation between them (between households), after adjusting for the study arm and study site.

In a model with no covariates, when analyzing protocol and supplement samples separately in mixed models, the ICCs for mothers and PM_2.5_ were 0.43 for protocol samples and 0.49 for supplemental samples. In models with no covariates, increasing the number of samples for mothers progressively from 2 (the first two postrandomization, which were 3 months apart) to 6, the within-person variance increased from 0.30 with two samples to 0.52 with all six, when first and last samples were 18 months apart. These trends for within-person variance were similar for mothers for BC and CO and for children.

In additional supplemental analyses, we predicted overall means of PM_2.5_ (on the log scale) across six samples for 251 control mothers who had all six protocol samples. The Pearson correlations between 15 possible pairs (out of six) of samples ranged from 0.24 to 0.47, with a mean of 0.36. We ran linear regression analyses and estimated the variance explained (*R*^2^) using the average of log PM_2.5_ across 6 samples as the outcome and, using a variable for study site as a predictor and as the second predictor, either one sample (6 combinations), the average of 2 samples (15 possible combinations), the average of 3 samples (20 possible combinations), or the average of 4 samples (15 possible combinations) as predictors. We then averaged the *R*^2^ across the combinations for each scenario. The average *R*^2^ by number of predictors were 0.47, 0.69, 0.81, and 0.90 for use of 1, 2, 3, or 4 individual measurements, respectively. While these results are encouraging in suggesting three samples might be sufficient (assuming the *R*^2^ of 0.80 is reasonably good), they depend on the specific sample size and amount of between and within household variance in our study and are not therefore generalizable to other studies.

## Discussion

We have shown that the estimate of a long-term average (16 months for mothers, 9 months for children) of personal exposure across measurements did not differ between our protocol samples and supplemental exposure samples measured in a subset of households of the HAPIN trial, except for CO among mothers, where supplemental samples were somewhat lower. This was not entirely unexpected but nonetheless reassuring for trial investigators, providing confidence that our basic sampling scheme was sufficient to characterize the effect of our intervention on exposures. These averages for protocol and supplemental samples might have varied given the high level of within-person variance, as there may have been potentially differing time-varying household behaviors in supplemental versus protocol samples, which were not controlled in our model. It should be noted that HAP exposures are on the high end of fine particulate exposure assessments. Our results, and the large within-person variance we see, likely depend on personal behavior, which may change daily or over a longer period for a variety of reasons, including the number of people in the household, who is cooking, seasonal changes, and changes in the house environment (e.g., ventilation, which itself may change with season, behavior). HAP exposures are also driven by short-term peak events (during cooking and other combustion activities) that introduce additional daily variability.

Our repeated exposure measurements of pregnant mothers and their infants were made at a time when day-to-day activities and behaviors would be expected to change over time with the gestational stage of pregnancy and infant development patterns (e.g., sleeping, crawling/walking) during the first year of life. Repeated measurements are likely to capture such variations.^13^ Little has been reported on how HAP exposures for these populations may change over time, although the GRAPHS trial in Ghana found that maternal CO exposures decreased during gestation for both control and intervention arms,^14^ a similar trend to those reported for HAPIN.^8^ The GRAPHS trial also documented temporal variability in PM_2.5_ exposures associated with the Harmattan season (blowing dust from the Sahara), further indicating that specific contextual (time-varying) factors should be taken into account when considering the number and timing of repeated samples required to sufficiently characterize long-term exposures. Here, we were able to account for time-varying variables for “winter” and “weekend”, while estimating mothers’ long-term average exposure, as well as the decrease in exposures for mothers after their child was born. The lower PM_2.5_ and BC exposures on weekends are notable as studies often do not measure during weekends due to logistical constraints, indicating the potential for bias when weekends are not included in the measurement schedule.

Mixed model prediction of exposures will, in general, provide estimates of long-term average exposure with reduced relative mean squared error (RMSE) compared to taking a simple arithmetic average of long-term exposure measures.^15,16^ Furthermore, others have suggested that the relative impact of additional measurements in accurately estimating long-term averages (over a period of one or two years) diminishes with more measurements. Keller and Clark^16^ used a different approach based on either 1) simulations with known true data to calculate long-term household averages, or 2) observed data from Honduras, where the “true” long-term household means for six samples were assumed to be those predicted from a mixed model. Their findings also suggest that three or four measurements over time may be optimal, although this will depend on the study-specific number of measurements and variances between and within households, as well as any local temporal trends. In our supplementary analyses of control mothers with complete protocol sampling data (6 repeat samples), in which we predicted individual mean exposure of log PM_2.5_ across all six samples, we found that the use of 1, 2, 3, or 4 samples predicted the overall mean with *R*^2^ of 0.51, 0.73, 0.81, and 0.90. These results suggest that, at least in our study, the use of 3 samples across our follow-up period of 16 months would provide a good estimate of long-term exposure (assuming an *R*^2^ of 80% is deemed adequate, which we believe is often the case, although admittedly there are no generally accepted guidelines for what level of *R*^2^ is acceptable). Our results depend on the overall sample size measured, the household variance, and the within-person correlation across measures (in our case averaging 0.36 for the Pearson correlation of pairs of log PM_2.5_ samples); these will, of course, vary among different study populations.

There is another advantage to repeated measurements on a subsample, assuming they are representative, besides assessing long-term average exposure. The intraclass correlation coefficient (ICC) in the subsample, calculated in a model without covariates, can be used to adjust for measurement error. In the case of repeated measures of exposure when the assumptions of (a) no systematic bias and (b) classical, nondifferential error if both hold, the ICC can be used to estimate the true exposure–response coefficient for health effects. This is done by dividing the observed exposure–response coefficient, derived by regressing the health effect on exposure (and potential confounders) using only the protocol samples by the ICC based on a subset where supplemental observations were taken (see Armstrong^17^ and Rosner et al.^18^ for the derivation of this relationship). This can be done even without the need for a true validation study to determine the relationship between measured and true exposure.

In summary, we found that doubling the number of exposure measurements in a 10% subsample of mothers and their infants did not appreciably change the overall average exposure estimates for either mothers or infants during the follow-up period of the HAPIN trial (although the precision of the estimate will, of course, improve with more samples). This finding is reassuring and suggests that the study’s sampling protocol was reasonably sufficient for accurately estimating long-term HAP exposures in our study populations. However, the importance of considering contextual factors (confounders which might be time-varying) and the potential impact of large within-person variance, as well as potential large measurement error, should not be discounted when determining the optimal number of measurements needed to accurately estimate long-term averages. Another consideration is whether to use resources to obtain more samples versus increasing measurement accuracy with high-quality instrumentation. Given the advances in lightweight and monitors for both PM_2.5_ and CO, we believe that investigators can now prioritize additional sampling (i.e., obtaining more samples per participant) while still collecting high-quality data.

## Conclusion

For our subsample, additional exposure measurements did not markedly change the average exposure estimation.

## Supporting Information Available

Table with results for children using mother’s measurements when child’s measurement missing, figures showing exposure contrasts for women in trial, and QQ plots comparing distributions of protocol and supplemental samples of PM_2.5_, BC, and CO for women and children (PDF)
