# Supplementary material for: Optimizing Exposure Measures in Large-Scale Household Air Pollution Studies: Results from the Multicountry HAPIN Trial
Source: Environ Sci Technol. 2025 Jan 14;59(3):1693–9. doi: 10.1021/acs.est.4c08052 (PMC11780738; doi:10.1021/acs.est.4c08052)

Supporting information

Optimizing exposure measures in large-scale household air pollution studies: results from the multi-country HAPIN trial.

Kyle Steenland*[1], Ajay Pillarisetti [2], Michael Johnson [3], Josh Rosenthal [4], Kalpana Balakrishnan [5], Lindsay Underhill [6], Lisa Thompson [7], John McCracken [8], Lance Waller [1], Laura Nicolaou [9], Maggie Clark [10], William Checkley [11], Jennifer Peel [10], Tom Clasen[1]

Supporting Information, 7 pages, 1 Table, 8 Figures

Table 1. Results for children using mother's PM.5 data when child's was missing
Figure 1. PM2.5 levels for mothers across six sampling times, for intervention and control groups
Figure 2. QQ plot for mothers, protocol vs supplemental samples, for PM2.5
Figure 3. QQ plot for infants, protocol vs supplemental samples, for PM2.5,
Figure 4. QQ plot for infants, protocol vs supplemental sampled, for ln(PM2.5)
Figure 5. QQ plot for mothers, protocol vs supplemental samples, for black carbon (BC)
Figure 6. QQ plot for mothers, protocol vs supplemental samples, for carbon monoxide (CO)
Figure 7. QQ plot for infants, protocol vs supplemental samples, for carbon monoxide (CO)
Figure 8. QQ plot for infants, protocol vs supplemental samples, for log carbon monoxide (CO)

Table 1. Child PM2.5 data imputing missing PM2.5 using mothers' PM2.5

Ln(PM2.5), n=1211**							
			coeff	std err	T value	P=value	ICC
Intercept			3.246	0.079	40.1	<.0001	0.40
country	Guatemala		0.070	0.097	0.73	0.45	
country	India		0.105	0.096	1.09	0.28	
country	Peru		-0.654	0.096	-6.83	<0.0001	
country	Rwanda		0				
supplemental			0.048	0.047	1.02	0.31	
s6_arm	Control	1	0.759	0.064	11.86	<.0001	
s6_arm	Intervention	0	0	.	.	.	
winter			0.135	0.055	2.45	0.01	
weekend			-0.138	0.142	-0.97	0.33	

*Log-transformed pollutants were regressed on an indicator variable for supplemental vs protocol samples and on study arm, study site, winter vs no winter, and weekend vs not weekend. Model ICC was 0.18.

** Number of valid measurements for the children who had both protocol and supplemental measurements.

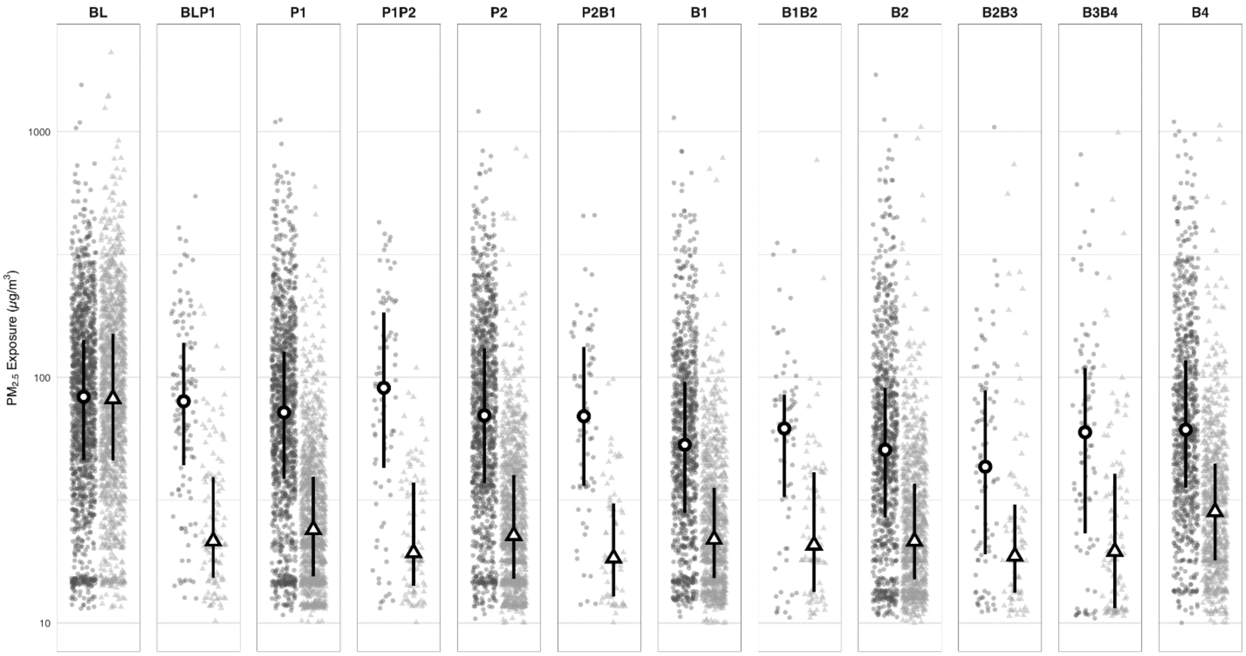

Figure 1. PM2.5 exposure of mothers over 16-month period, including supplemental sampling. Panels are in order from first (baseline) sample till the last sample at the end of the first year of the child. BL to P2 are during pregnancy, and B1 to B4 are during the first year of the infants' life. BLP1, P1P2, P2B1, B1B2, B2B3, and B3B4 are supplemental samples taken between the protocol samples. In each panel the shaded dots on the left are the control arm, while those on the right are the intervention arm.

Figure 2. QQ plot for PM2.5, mothers

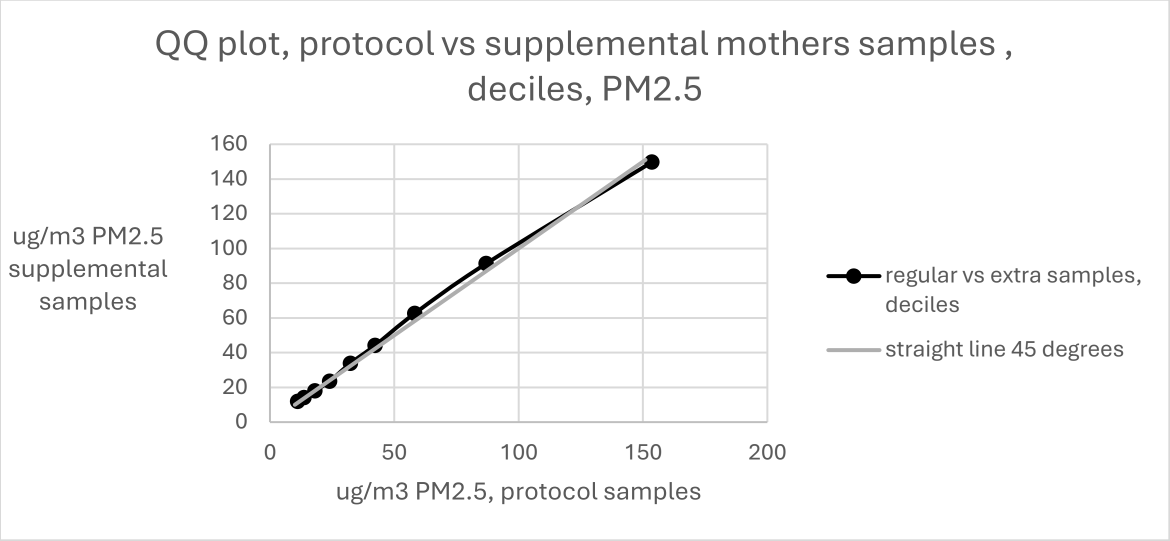

Figure 3. QQ plot for PM2.5, infants

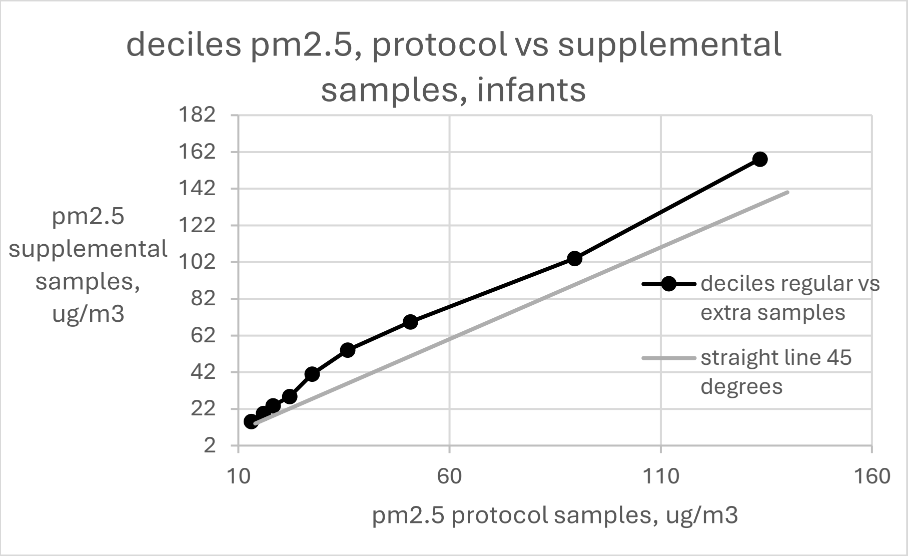

Figure 4. QQ plot for ln(PM2.5), infants

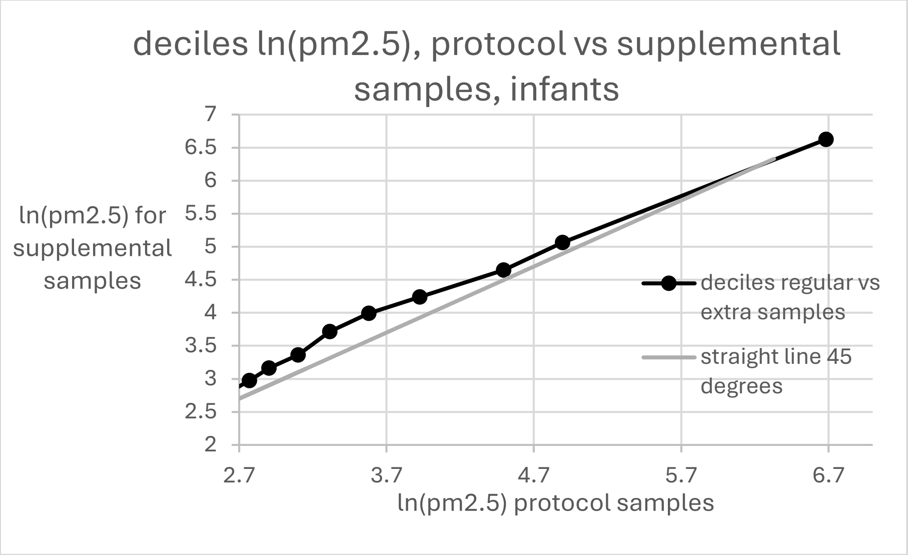

Figure 5. QQ plot for mothers, protocol vs supplemental samples, for black carbon (BC)

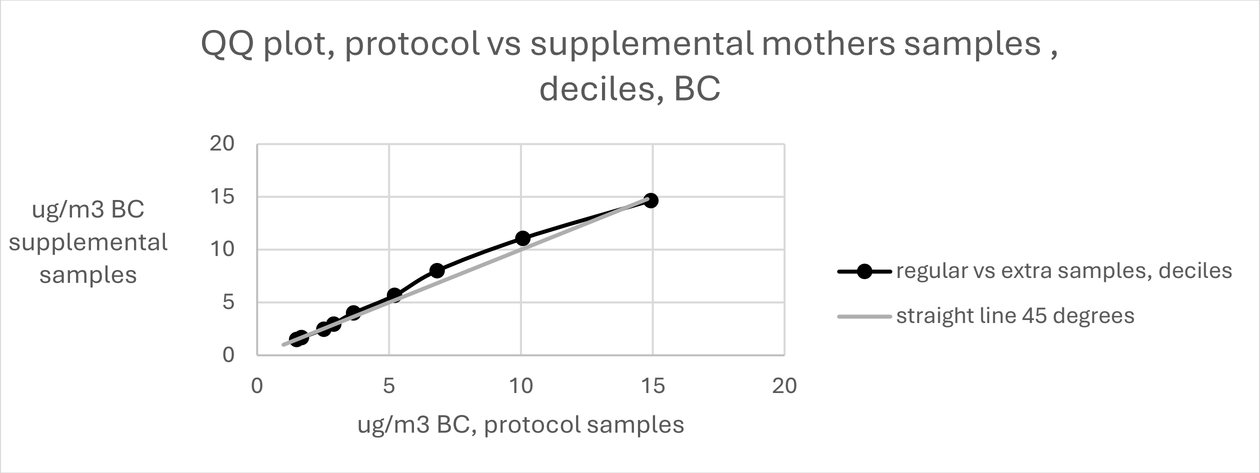

Figure 6. QQ plot for mothers, protocol vs supplemental samples, for carbon monoxide (CO)

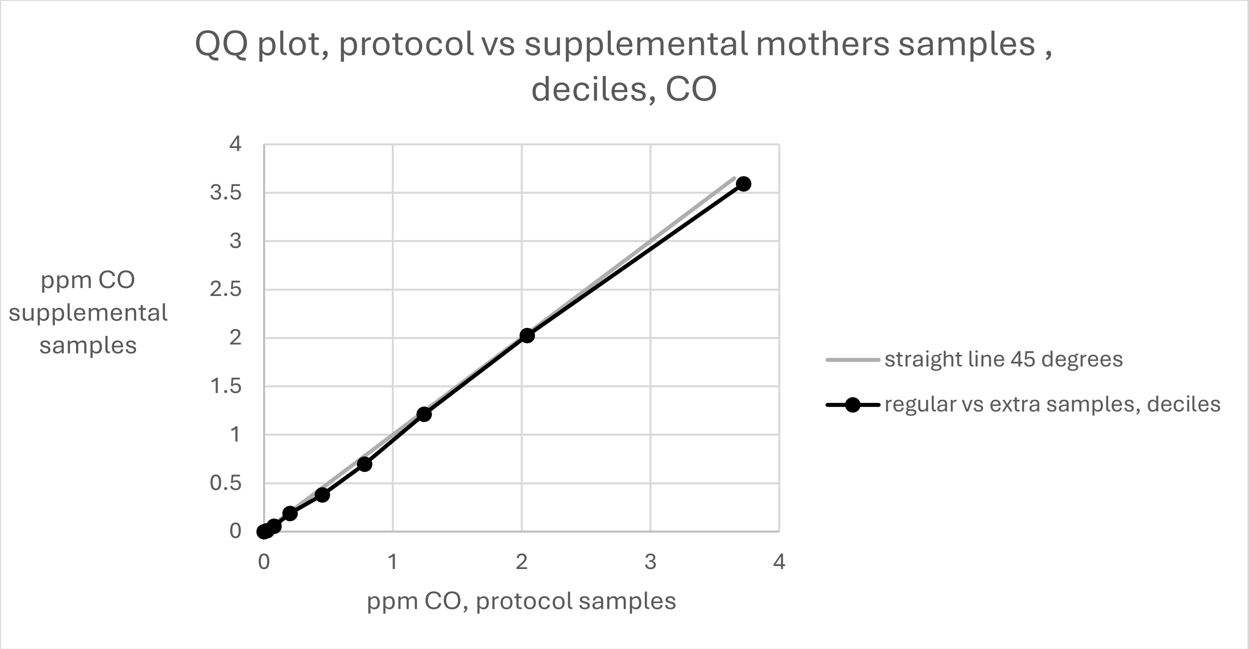

Figure 7. QQ plot for infants, protocol vs supplemental samples, for carbon monoxide (CO)

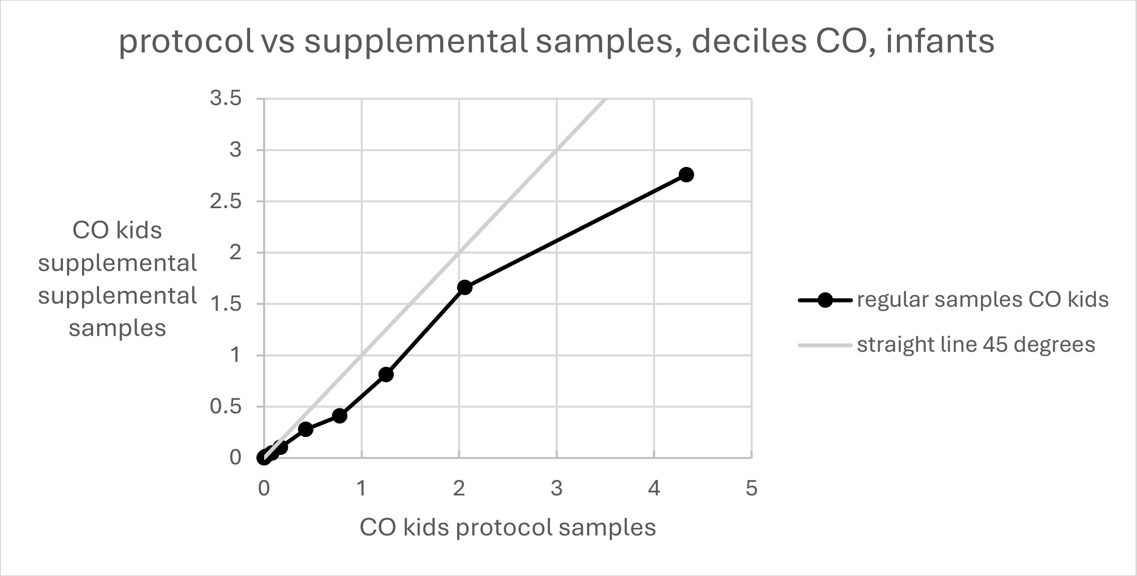

Figure 8. QQ plot for infants, protocol vs supplemental samples, for log carbon monoxide (CO)

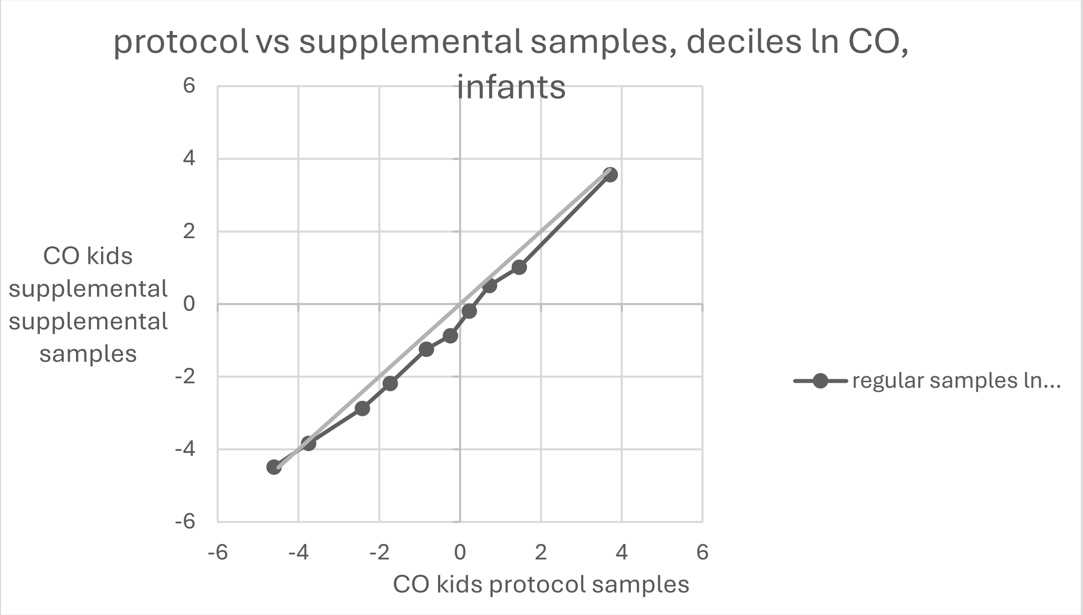

Supplement: Supplementary file 1 — es4c08052_si_001.pdf [file es4c08052_si_001.pdf]
